# Influencing Factors and Risk Assessment of Precipitation-Induced Flooding in Zhengzhou, China, Based on Random Forest and XGBoost Algorithms

**DOI:** 10.3390/ijerph192416544

**Published:** 2022-12-09

**Authors:** Xun Liu, Peng Zhou, Yichen Lin, Siwei Sun, Hailu Zhang, Wanqing Xu, Sangdi Yang

**Affiliations:** 1 School of Arts and Communication, China University of Geosciences (Wuhan), Wuhan 430070, China; 2 School of Civil Engineering and Architecture, Wuhan Institute of Technology, Wuhan 430074, China

**Keywords:** precipitation-induced flooding, influencing factors, risk assessment, random forest, XGBoost

## Abstract

Due to extreme weather phenomena, precipitation-induced flooding has become a frequent, widespread, and destructive natural disaster. Risk assessments of flooding have thus become a popular area of research. In this study, we studied the severe precipitation-induced flooding that occurred in Zhengzhou, Henan Province, China, in July 2021. We identified 16 basic indicators, and the random forest algorithm was used to determine the contribution of each indicator to the Zhengzhou flood. We then optimised the selected indicators and introduced the XGBoost algorithm to construct a risk index assessment model of precipitation-induced flooding. Our results identified four primary indicators for precipitation-induced flooding in the study area: total rainfall for three consecutive days, extreme daily rainfall, vegetation cover, and the river system. The Zhengzhou storm and flood risk evaluation model was constructed from 12 indicators: elevation, slope, water system index, extreme daily rainfall, total rainfall for three consecutive days, night-time light brightness, land-use type, proportion of arable land area, gross regional product, proportion of elderly population, vegetation cover, and medical rescue capacity. After streamlining the bottom four indicators in terms of contribution rate, it had the best performance, with an accuracy rate reaching 91.3%. Very high-risk and high-risk areas accounted for 11.46% and 27.50% of the total area of Zhengzhou, respectively, and their distribution was more significantly influenced by the extent of heavy rainfall, direction of river systems, and land types; the medium-risk area was the largest, accounting for 33.96% of the total area; the second-lowest-risk and low-risk areas together accounted for 27.09%. The areas with the highest risk of heavy rainfall and flooding in Zhengzhou were in the Erqi, Guanchenghui, Jinshui, Zhongyuan, and Huizi Districts and the western part of Xinmi City; these areas should be given priority attention during disaster monitoring and early warning and risk prevention and control.

## 1. Introduction

Precipitation-induced flooding refers to the intense accumulation of precipitation, rising or overflowing water levels in rivers, lakes, and reservoirs, and the inability of water to drain away due to heavy or continuous rainfall [1]; this can lead to low-lying houses and farmland becoming waterlogged or inundated, causing significant economic losses and endangering lives [2]. As a result of extreme weather phenomena exacerbated by global climate change, precipitation-induced flooding has become one of the most frequent, widespread, and destructive natural disasters around the world [3].

From 19 to 23 July 2021, Henan Province in central China experienced anomalously heavy rainfall, with precipitation of >400 mm at 43 observation stations, >300 mm at 154 stations, >200 mm at 467 stations, and >100 mm at 1426 stations. During that time, 19 cities and counties in the province broke their daily precipitation records. Zhengzhou, the provincial capital, which is located in a flood disaster zone, experienced a total rainfall of 993.1 mm and a cumulative surface rainfall of 543 mm during the event [4,5]. The city broke 70-year records for hourly and daily precipitation since the establishment of the first meteorological station in Zhengzhou in 1951. The economic losses and human casualties caused by the floods were considerable [5]. According to data released by the Chinese Ministry of Emergency Management, the floods affected 14.786 million people in 150 counties, county-level cities, and districts in Henan Province and killed 398 people, including 380 (95.5%) people in Zhengzhou [6]. The economic losses sustained by the province amounted to RMB 120.06 billion, 34.1% of which (RMB 40.9 billion) was in Zhengzhou.

Risk assessments of precipitation-induced flooding can be conducted by analysing the influencing natural [7], geographical, social, economic, population, and industrial factors in the study area [8]. The construction of a model to assess the risk of regional precipitation-induced flooding allows the identification of areas at high risk of such events within a specific area, which can be made the focus of disaster monitoring and warnings as well as risk prevention and control work, which is vital for regional economic development and social stability [9].

The monitoring and early warning of storm and flood disasters and risk management have been a common concern in the world for nearly half a century; many breakthroughs have been made in domestic and international research on storm and flood disaster risk assessment. Regarding the chronological order of application scenarios, existing storm and flood hazard assessments can be divided into three categories: pre-disaster assessments [10], mid-disaster follow-up monitoring and assessments, and post-disaster real-world assessments; in terms of research scales, there are studies that consider large areas and watersheds as units for overall regional risk assessment and centralised control [11]; there are also studies that consider the differences in the basic characteristics of specific cities [12,13]. From the perspective of research methods, most of the existing studies are based on statistical principles combined with 3S technologies [14,15], including hierarchical analysis, entropy value method, logistic regression method [16], BP neural network evaluation method, intelligent algorithms combined with RS-GIS technology [17,18], and hydrodynamic models [19,20], etc.; from the selected index system, the 10 index factors of topographic index, water system index, number of consecutive rainstorm days, 1 h rainfall, 24 h cumulative rainfall, land-use type, vegetation cover, population density, old and young population ratio, and GDP were used with the highest frequency and recognition. Overall, China has developed a mature system for assessing flood risks, which has been applied widely in regional flood monitoring and early warning systems as well as in risk prevention and control. Nevertheless, probing deeper into the existing literature on risk assessments of precipitation-induced flooding, we found two areas that require further attention. First, the index systems used are too similar and do not give sufficient consideration to regional attributes. The assessment indicators chosen in most studies are based on previous studies, which ignores the fact that the contribution rates to flooding of various indicators depend on the geographical, climatic, and socio-economic environments of a particular area. Second, regarding methods of calculating indicator weights, subjective weighting methods (such as the analytic hierarchy process [21]) rely too heavily on individual judgements, and objective weighting methods (such as entropy methods) are hampered by missing data, leading to questionable results.

To overcome subjectivity and lessen the influence of missing data, the random forest (RF) and XGBoost algorithms can be used to select the index system as well as to calculate index weights for the risk assessment of precipitation-induced flooding. RF has been widely used in research on geological disasters, such as landslides and debris flows, as well as on air pollution [22]. It can calculate the contribution to the final risk of each factor in the index, which can be used to screen and optimise flooding assessment indicators. XGBoost can determine a default direction of the branch for missing data, thereby reducing the resulting error, and it can handle both classification and numerical features with better inclusiveness, stability, and accuracy [23].

To assess the precipitation-induced flooding that occurred in the city of Zhengzhou in the central Chinese province of Henan in July 2021, we chose 16 basic indicators, including elevation, slope, river systems, extreme daily rainfall, total rainfall over three consecutive days, and vegetation cover. We used RF to examine the contributions of each of the indicators to precipitation-induced flooding and then screened and optimised the indicators based on the area under the curve (AUC) and accuracy (ACC) of the RF model to create our risk-assessment index. We then introduced the XGBoost [24] algorithm to construct a risk index assessment model to identify high-risk zones of the city [25]. This study is expected to serve as a scientific basis for precipitation-induced flooding prevention and mitigation planning in Zhengzhou.

## 2. Materials and Methods

### 2.1. Overview of the Study Area

**Figure 1:**
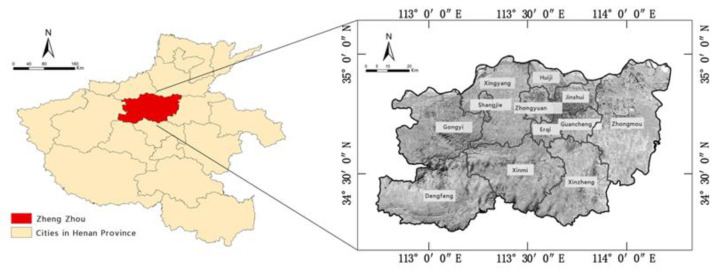
Map of the study area.

The city of Zhengzhou is located in the north-central part of Henan Province in the lower reaches of the Yellow River [26]. It has been designated by the State Council as an important core city in central China and a major national transportation hub [27]. Zhengzhou comprises six municipal districts, five county-level cities, and one county [28], as shown in Figure 1. It has a northern temperate continental monsoon climate, with an average annual rainfall amount of 640.9 mm and precipitation levels that, in general, decrease in a south-to-north direction. Its terrain is higher in the southwest and lower in the northeast. The city contains a complex system of 124 rivers of various sizes [29]; it is intersected by the two major river systems of the Yellow River and the Huai River. The other main waterways in Zhengzhou are the Suoxu River, Wei River, Jialu River, Jinshui River, Xiong’er River, Qili River, and the Dongfeng Canal [30].

### 2.2. Research Methods

#### 2.2.1. GIS-Weighted Integrated Evaluation Method

In accordance with natural disaster risk-assessment theory [18,30], this study first assessed the risk posed by each of the four aspects of flood-aggravating environmental susceptibility, flood-causing risk, location-specific exposure, and flood-mitigation capability, which provided a multi-dimensional assessment of the occurrence and influencing factors of precipitation-induced flooding in Zhengzhou. A weighted comprehensive evaluation method was used to assess the four aspects. The equation is as follows:

(1) $$G=\overset{n}{\underset{i=1}{\sum }}W_{i}\times D_{i\;},\;$$

where G is the overall index of each individual assessment, n is the number of indicators, $W_{i}$ is the weight of each indicator in the final risk, and $D_{i}$ is the value of each indicator after normalisation.

Based on the principles of disaster risk assessments, after obtaining the individual assessment index of flood-aggravating environmental susceptibility, flood-causing risk, location-specific exposure, and flood-mitigation capability, we used the power-index-weighted assessment method to integrate and overlay the individual assessment results and establish a model for assessing the risk of precipitation-induced flooding in Zhengzhou:

(2) $$FDRI=f\left(VH,VE,VS,VR\right),$$

where FDRI is the final result of the flood risk assessment; f is the power-index model; and VH, VE, VS, and VR are the susceptibility, risk, exposure, and mitigation capability assessment indicators calculated using Equation (8).

#### 2.2.2. Random Forest

RF is an ensemble learning method that constructs a multitude of decision trees [31]. It uses bootstrap resampling to select samples with the same number of features from the original training dataset [32]. Decision trees are then built for each sample, and predictions of multiple decision trees are combined to obtain the final result by voting or averaging.

RF uses the Gini Index (GI) for importance weighting and has been used widely in existing research to evaluate feature importance. This is determined by averaging the change in the GI for each feature at each decision tree node split and presenting those data as a percentage of the total average GI changes of all features.

The feature importance score is denoted by the variable importance measure (VIM), and the Gini Index is denoted by GI [33]. Assuming there are m features $(X_{1},\;X_{2},\;X_{3}$, …, $X_{m})$, the first step is to calculate the GI score $VIM_{j}^{\left(Gini\right)}$ of each feature $X_{j}$. The j-th feature is the average change in impurity of the node splits for all decision trees [34]. The equation is as follows [35]:

(3) $$GI_{m}=\overset{k}{\underset{k=1}{\sum }}\underset{k^{\prime }}{\sum }p_{mk}p_{{mk}^{\prime }}=1-\overset{\left|k\right|}{\underset{k=1}{\sum }}p_{mk}^{2},\;$$

(4) $$VIM_{jm}^{\left(Gini\right)}=GI_{m}-GI_{l}-GI_{r},\;$$

where k is the number of categories, $p_{mk}$ is the proportion of category k in node m, $VIM_{jm}^{\left(Gini\right)}$ is the change in the GI of feature $X_{j}$ when node m splits, and $GI_{l}$ and $GI_{r}$ are the GIs of the two new nodes after the branch [34].

If the node of feature $X_{j}$ in decision tree i is set as M, the importance of $X_{j}$ in tree i is:

(5) $$VIM_{jm}^{\left(Gini\right)}=\underset{m\in M}{\sum }VIM_{jm}^{\left(Gini\right)}.\;$$

If there are n trees in the RF, the sum of the GI changes of feature $X_{j}$ in all decision trees is [36,37]:

(6) $$VIM_{j}^{\left(Gini\right)}=\overset{n}{\underset{i=1}{\sum }}VIM_{ij}^{\left(Gini\right)}.\;$$

Finally, normalisation is performed to obtain the feature importance of $X_{j}$:

(7) $$VIM_{j}=\frac{VIM_{j}}{\sum_{i=1}^{c}VIM_{i}}.\;$$

The accuracy of RF is higher than that of a single algorithm due to it being an ensemble algorithm [38]. Bootstrap resampling greatly increases the randomness of the training dataset and corrects for the habit of overfitting, thereby improving stability. It is considered one of the best machine learning models.

#### 2.2.3. XGBoost

Extreme gradient boosting (XGBoost) [24] is an efficient gradient-boosting decision tree algorithm that can be used to calculate index weights [39]. XGBoost continuously adds trees and splits features to grow a tree. Each time a tree is added [40], a new function is learned, and each round of prediction is used to fit the residual from the previous round of prediction [41]. The score of a sample can be predicted based on the features of the sample. When the training is complete, there are *n* trees. Each tree will fall to a corresponding leaf node, and each leaf node corresponds to a score. Finally, the corresponding scores of all trees are added to obtain the predicted value of the sample. The equation is as follows:

(8) $$\hat{y}=\emptyset \left(x_{i}\right)=\overset{k}{\underset{k=1}{\sum }}f_{k}\left(x_{i}\right),\;$$

(9) $$where\;F=\left\{f\left(x\right)=\omega_{q\left(x\right)}\right\}\left(q:R^{m}\to T,\omega \in R^{T}\right),\;$$

where $\omega_{q\left(x\right)}$ is the score of leaf node q, and $f\left(x\right)$ is a regression tree.

In the XGBoost algorithm, there are strong correlations between successive decision trees [42]. Each round of prediction is based on the prediction error in the previous round; thus, it is iteratively constructed, which greatly improves the accuracy of the prediction [43]. Compared with traditional statistical models, it can determine a default direction of a branch for missing data, thereby reducing the resulting error [44]. It can also handle both categorical and numerical features, giving the prediction model greater stability.

#### 2.2.4. Accuracy and the Area under the Curve

ACC [45] uses a test set to classify the model, with the proportion of correctly classified records out of the total number of records used to judge the quality of the classification results [46].

AUC is the area under the receiver operating characteristic curve enclosed by the coordinate axis [19,20]. Its value range is usually 0.5–1, and it is commonly used to measure the prediction performance of machine learning. The closer the AUC is to 1, the higher the prediction accuracy of the model. When the AUC is close to 0.5, it indicates that the model has no practical application value.

Using a combination of ACC and AUC to measure the stability and accuracy of a model can avoid erroneous results caused by skewed data that often occur in data samples [14].

### 2.3. Data Sources

#### 2.3.1. Flooded Areas

**Figure 2:**
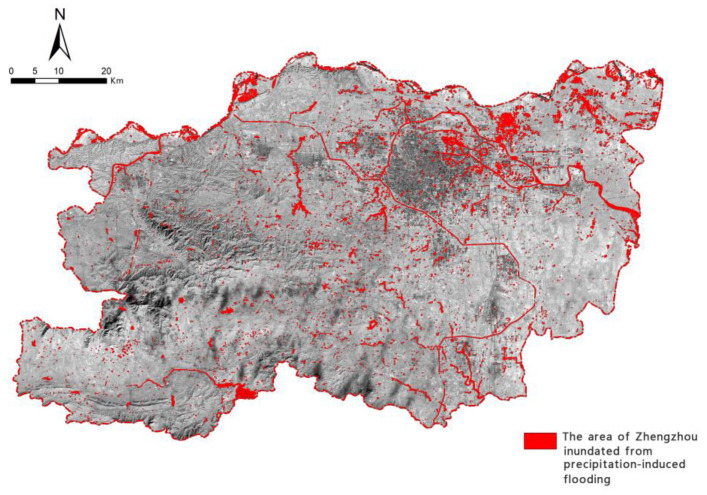
The area of Zhengzhou inundated from precipitation-induced flooding.

The Sentinel-2 high-resolution, multispectral imaging products used in this study were downloaded from the Copernicus Open Access Hub (https://scihub.copernicus.eu/) (accessed on 24 November 2022). Due to the influences of cloud cover and temporal resolution [47], post-flood images of Zhengzhou were created using Sentinel-2 images from 19 to 25 July 2021, and pre-flood images [48] of Zhengzhou were created using Sentinel-2 images from 15 to 27 April 2021. The data product was Level-1C, and the spatial resolution was up to 10 m. After the pre-processing steps of radiometric calibration, geometric correction, and atmospheric correction were performed on the data, SNAP and ENVI software were used to change the data format. Using the pre- and post-flood images [49], three region-of-interest samples were identified: original water bodies, flooded areas, and land areas [50]. The graph-based segmentation algorithm was used to conduct supervised classification with comparative experiments [51]. The results were then subjected to primary/secondary analysis and clustering post-processing as well as manual correction to obtain the flooded area of Zhengzhou during the precipitation-induced flooding in July 2021, as shown in Figure 2.

#### 2.3.2. Assessment Indicators

According to traditional risk-assessment theory, regional precipitation-induced flooding is predominantly caused by meteorological factors, i.e., extreme rainfall, together with a combination [52] of geo-environmental factors and the vulnerability of the affected location [53]. Many recent studies have demonstrated [54] that disaster prevention and mitigation efforts, such as early warning systems, drainage infrastructure, rescue services, publicity and education, and emergency shelters to protect against precipitation-induced flooding are important factors in evaluating a region’s risk of flooding. Thus, based on our evaluation of the geographical features of Zhengzhou and the availability of data, and after conducting correlation testing [55] and analysis, we selected the following 16 indicators across the four categories of flood-aggravating environmental susceptibility, flood-causing risk, location-specific exposure, and flood-mitigation capability [56]: elevation, slope angle, slope aspect [57], river system, roads, extreme daily rainfall, total rainfall over three consecutive days, night-time light brightness [58], land-use type, proportion of arable land, gross regional product (GRP), GDP per capita, economic growth rate, proportion of the elderly population, vegetation cover, and medical rescue services.

Night-time light brightness is a new and popular area of research. It uses the brightness of various lights at night to identify built-up areas and determine population distributions. It has been used in fields such as regional economics and urbanisation [59], but it has not been widely applied in natural disaster risk assessments. This study uses it to indicate the population density and urbanisation level of Zhengzhou, thereby avoiding the excessively high correlations of various socio-economic factors in traditional disaster assessments. Moreover, the raster data have a higher resolution than socio-economic panel data do.

The data sources of all the indicators are shown in Table 1.

Given the different sources and formats of the geographic, meteorological, and socio-economic data, we used ArcGIS software to transform the projections and resample the raster data in order to obtain unified coordinates and a resolution of 30 m that would allow analysis and calculations; this also ensured the accuracy of our assessments. Discrete data were spatially processed using kriging interpolation and converted into raster data with a resolution of 30 m.

## 3. Results

### 3.1. Flood Risk Assessment Index Analysis and Optimisation

#### Analysis of Indicator Contributions Using RF

**Figure 3:**
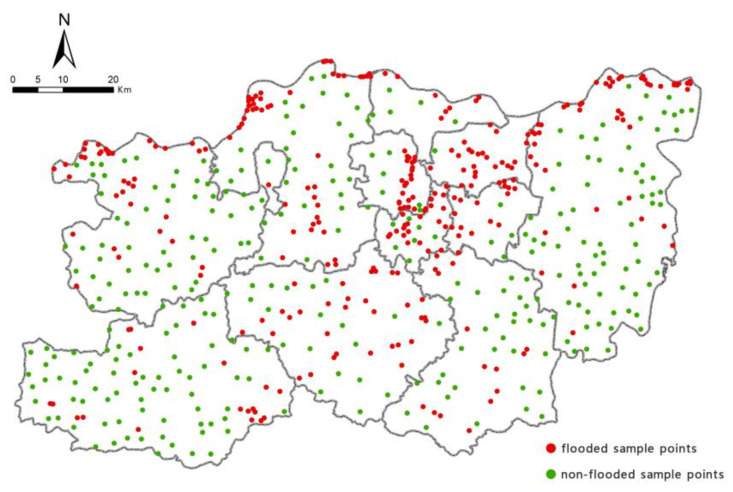
Distribution of sample points.

**Figure 4:**
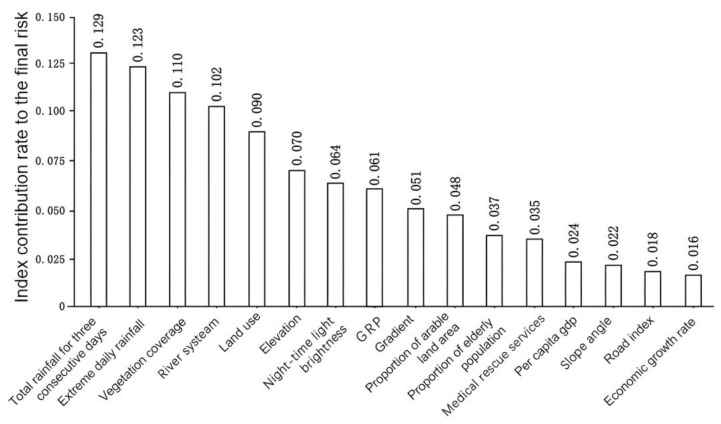
Ranked contribution rates of assessment indicators.

To guarantee the integrity of the samples, 300 flooded sample points were randomly selected from the extracted flooded areas of Zhengzhou and numbered ‘1’. Then, 300 non-flooded sample points were randomly selected and numbered ‘0’. The spatial distribution of the 600 sample points is shown in Figure 3. We entered the sample data into the RF model, with 70% of the sample points (i.e., 210 flooded sample points and 210 non-flooded sample points) set to be randomly selected, and the remaining 30% of sample points (i.e., 90 flooded sample points and 90 non-flooded sample points) set as the validation set. We ran the RF model using Python, with feature importance analysis conducted for the 16 indicators. The main parameters of the RF model were as follows: n_estimators = 100, criterion = ‘gini’, max_depth = ‘None’, min_samples_split = 2, min_samples_leaf = 20, max_features = ‘sqrt’, min_impurity_decrease = 0.0, bootstrap = True, oob_score = True, n_jobs = 1, random_state = None. The ranked contributions of the assessment indicators to precipitation-induced flooding in Zhengzhou are shown in Figure 4.

Of the 16 assessment indicators, 4 had a contribution rate to the final risk that was greater than 0.100. In descending order, these were total rainfall over three consecutive days (0.129), extreme daily rainfall (0.123), vegetation cover (0.110), and river systems (0.102). The contribution rates of total rainfall over three consecutive days and extreme daily rainfall were the highest, indicating that sustained and high-volume cumulative rainfall as well as extremely heavy short-term rainfall are the main factors in Zhengzhou that lead to precipitation-induced flooding [60]. Vegetation cover can delay and reduce the level of the flood peak by affecting surface runoff during rainfall, so its contribution rate was the third highest. The contribution rate of the river system indicator was 0.102, which demonstrates that areas with precipitation-induced flooding in Zhengzhou are closely coupled with the distribution of the city’s river system.

Five indicators had contribution rates between 0.050 and 0.100. In descending order, they were land-use type (0.090), elevation (0.070), night-time light brightness (0.064), GRP (0.061), and slope angle (0.051). The higher contribution of land-use type reflects the close relationship between land-use type and the spatial distribution of areas prone to precipitation-induced flooding. The contribution rates of the elevation and slope angle reflect the fact that topography plays a notable promotional role in precipitation-induced flooding. The contributions of night-time light brightness and GRP indicate that the spatial distribution, development, and migration of the urban population, economy, and industry have a direct bearing on the spatial locations affected by precipitation-induced flooding.

Three indicators had contribution rates between 0.025 and 0.050: proportion of arable land (0.048), proportion of the elderly population (0.037), and medical rescue services (0.035). Arable land and the elderly population are both vulnerable to floods, and medical rescue services are an important indicator of regional disaster prevention and mitigation capabilities.

Four indicators had contribution rates lower than 0.025: per capita GDP (0.024), slope aspect (0.022), roads (0.018), and economic growth rate (0.016). This means that the correlations between these indicators and the final risk of precipitation-induced flooding in Zhengzhou were relatively weak.

### 3.2. Optimisation of Flood Risk-Assessment Indicators

#### Optimisation of Assessment Indicators

Based on the ranking of contribution rates to the final risk, starting with the indicator with the lowest contribution rate, we removed assessment indicators sequentially to construct the experimental model. A new model was constructed each time an indicator was removed. We retained the same sample numbers and ratio of the training set to the validation set and used Python to implement each experimental model in turn. The accuracy and stability of each model were determined based on the AUC and ACC values, leading to the optimal model structure. A total of eight experiments were set up, and the results were recorded in Table 2.

According to the experimental process records, the ACC and AUC in the experimental model first increased and then decreased. In Experiments 1 to 5, the removal of indicators with small contribution rates reduced the disruptive data in the model, which continuously streamlined and optimised the model structurally. As a result, the model’s accuracy and stability improved, with ACC and AUC values increasing significantly. In Experiments 6 to 9, however, as indicators with larger contribution rates were removed, the effective data in the model decreased, which affected the model’s performance and caused ACC and AUC values to gradually decrease. It can be seen from Table 2 that ACC values peaked in Experiment 5, increasing from 88.0% in Experiment 1 to 91.3%, whereas AUC values peaked in Experiments 5 and 6, with a value of 0.967. The latter values were close to 1, indicating that the model’s predictions were highly accurate. Looking at both the ACC and AUC values, the optimal model structure was that in Experiment 5, in which the four indicators of economic growth, roads, slope aspect, and per capita GDP were removed.

**Figure 5:**
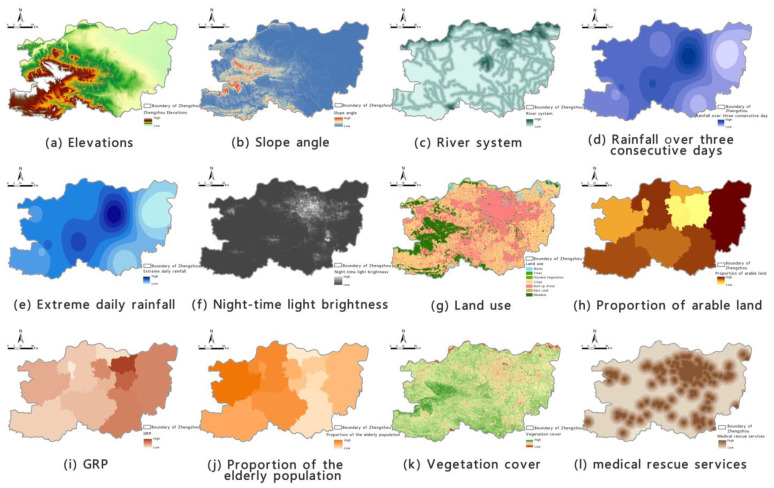
Diagrams of individual precipitation-induced flooding risk-assessment factors.

RF was used to analyse the contribution rate of the 16 indicators, with the model prediction performance and accuracy judged using AUC and ACC values as various indicators were compared and removed. This ultimately led us to the optimal precipitation-induced flooding assessment index based on local conditions in Zhengzhou. The optimal index system consists of 12 indicators: elevation, slope angle, river system, extreme daily rainfall, total rainfall over three consecutive days, night-time light brightness, land use, proportion of arable land, GRP, proportion of the elderly population, vegetation cover, and medical rescue services. Each assessment indicator after normalisation is shown in Figure 5.

### 3.3. Precipitation-Induced Flooding Risk Assessment

#### Assessment Indicator Weighting

We calculated the weight of each of the 12 assessment indicators selected above relative to the final risk to construct the risk-assessment model of precipitation-induced flooding in Zhengzhou. To overcome the issue of excessive subjectivity in the weighting calculation methods of traditional disaster risk assessments as well as to minimise the impact of missing and misattributed data, we used XGBoost to calculate the weights of each assessment indicator. The model’s main parameters were set as follows: n_estimators = 91, max_depth = None, min_samples_leaf = 13, min_samples_split = 2, max_features = ‘auto’, bootstrap = True, oob_score = False, n_jobs = 1, random_state = 10. Using Python to implement the XGBoost model, we obtained the weight of each assessment indicator (as shown in Table 3).

### 3.4. Assessment Results and Analysis

#### 3.4.1. Analysis of Individual Assessment Results

**Figure 6:**
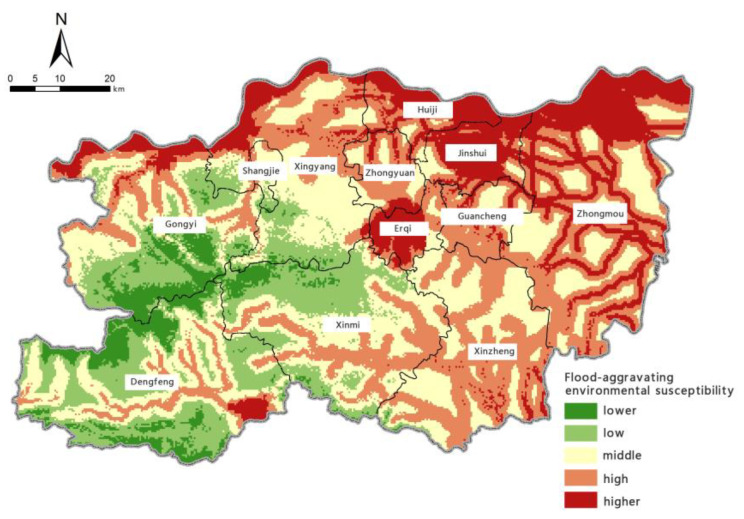
Zoning map of flood-aggravating environmental susceptibility.

**Figure 7:**
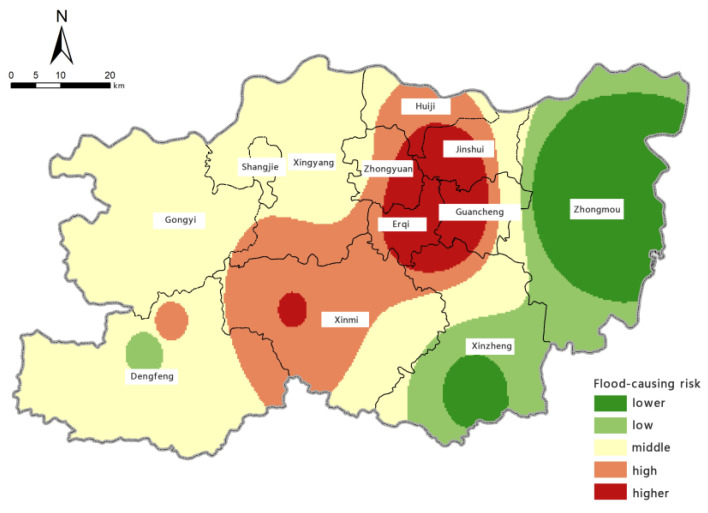
Zoning map of flood-causing risk.

**Figure 8:**
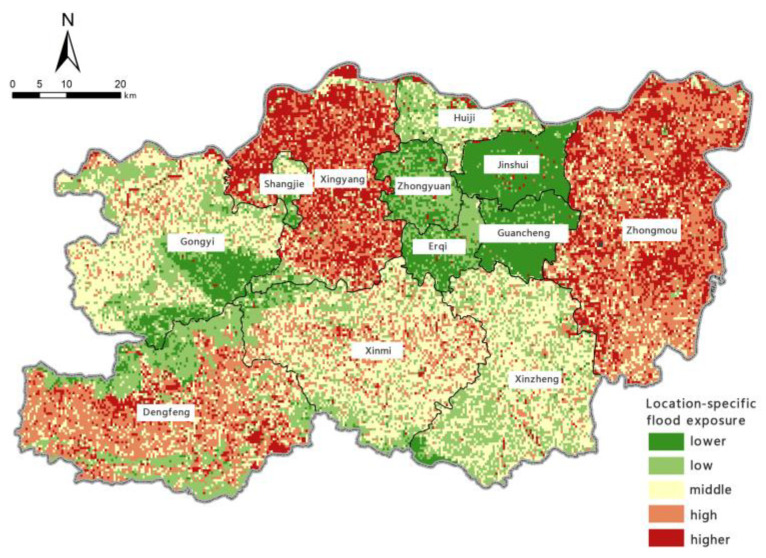
Zoning map of location-specific flood exposure.

**Figure 9:**
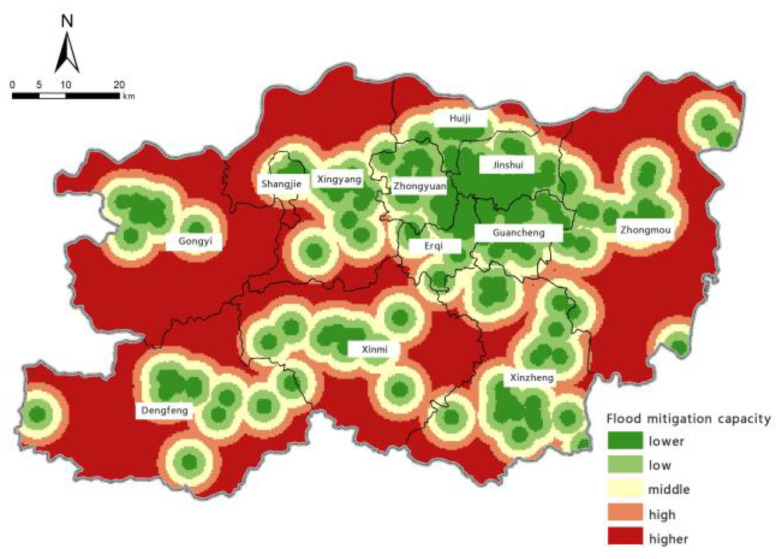
Zoning map of flood-mitigation capacity.

Based on the model for assessing the risk of precipitation-induced flooding outlined above, with the help of the spatial overlay analysis function in ArcGIS, we calculated the assessment index of flood-aggravating environmental susceptibility, flood-causing risk, location-specific exposure, and flood-mitigation capability for precipitation-induced flooding in Zhengzhou. Borrowing the five levels of risk classification used in previous studies [61], we used the natural breaks method to perform classifications. The zoning maps for each individual assessment aspect are shown in Figure 6, Figure 7, Figure 8 and Figure 9.

The zoning map of flood-aggravating environmental susceptibility shows that Zhengzhou’s susceptibility to precipitation-induced flooding is most significantly affected by its river system. Areas of ‘very high’ and ‘high’ susceptibility are mainly distributed along the northern Yellow River system, Baisha Reservoir, Jiangang Reservoir, and other secondary tributaries of the Yellow and Huai River systems. Areas with very high susceptibility are mainly in Huiji District, Jinshui District, Erqi District, Zhongmu County, near the Yellow River north of Xingyang City and Gongyi City, and around Baisha Reservoir in the southeast of Dengfeng City. Southwest Zhengzhou has relatively high terrain and steep slopes, which facilitate the discharge of floodwater. As a result, areas with ‘low’ and ‘very low’ susceptibility are mainly distributed in Dengfeng City, Gongyi City, Xinmi City, and the southwestern part of Xingyang City. The northern, eastern, and central parts of Zhengzhou have high flood-aggravating environmental susceptibility due to their flat terrain and limited drainage capacity.

It can be seen from the zoning map of flood-causing risk levels that areas with ‘very high’ and ‘high’ risks of precipitation-induced flooding in Zhengzhou are concentrated in Erqi District, Guancheng Hui District, Jinshui District, Zhongyuan District, Huiji District, and Xinmi City. Erqi District, the western parts of the Guancheng Hui and Jinshui districts, and the eastern part of Zhongyuan District are the most at risk; this result is closely linked to the distribution of heavy rainfall. Xingyang City, Shangjie City, Gongyi City, and Dengfeng City are in areas of moderate risk. Zhongmu County and Xinzheng City have the lowest risk of precipitation-induced flooding.

The zoning map of location-specific exposure to precipitation-induced flooding shows that areas with ‘high’ and ‘very high’ exposure are in Xingyang City, Dengfeng City, and Zhongmu County. Areas with moderate location-specific exposure are mainly in Gongyi City and Shangjie District in the northwest, Xinmi City and Xinzheng City in the south, and Huiji District in the north. The Central Plains District, Erqi District, Guancheng Hui District, and Jinshui District in the north-central area have the lowest exposure. Location-specific exposure is affected by population, economic, industrial, and resource factors. Xingyang City, Dengfeng City, and Zhongmu County have developed arable farming, so they have large proportions of arable land, which is less exposed to flooding; however, they are economically disadvantaged, as agricultural land has poor resilience to heavy rainfall and flooding, and it takes that type of land a long time to recover. Zhongyuan District, Erqi District, Guancheng Hui District, and Jinshui District have high night-time light brightness, indicating a large range and intensity of anthropogenic activities, so their population exposure is high, but because their proportions of built-up land are high, their land exposure is low. The location-specific exposure index is calculated based on the weights of the various factors, and the exposure levels of Zhongyuan District, Erqi District, Guancheng Hui District, and Jinshui District were found to be ‘low’ and ‘very low’.

It can be seen from the zoning map of flood-mitigation capability levels that Shangjie District, Zhongyuan District, Erqi District, Guancheng Hui District, Jinshui District, and other places near the main central urban area have the highest mitigation capability. Areas with ‘high’, ‘moderate’, ‘low’, and ‘very low’ levels of mitigation capability are distributed in descending order as one moves away from areas with the highest mitigation capabilities. An area’s flood-mitigation capability is determined by factors including infrastructure, natural conditions, and social welfare, and it is manifested as resistance to flooding and resilience in recovery. This study used medical rescue services as an assessment indicator. The closer a location is to the city centre and the main urban area, the higher the density of large-scale medical institutions, such as general hospitals and 3A hospitals (the highest classification in China), and, thus, the stronger its ability to mitigate the danger posed by disasters.

#### 3.4.2. Analysis of Assessment Results

**Figure 10:**
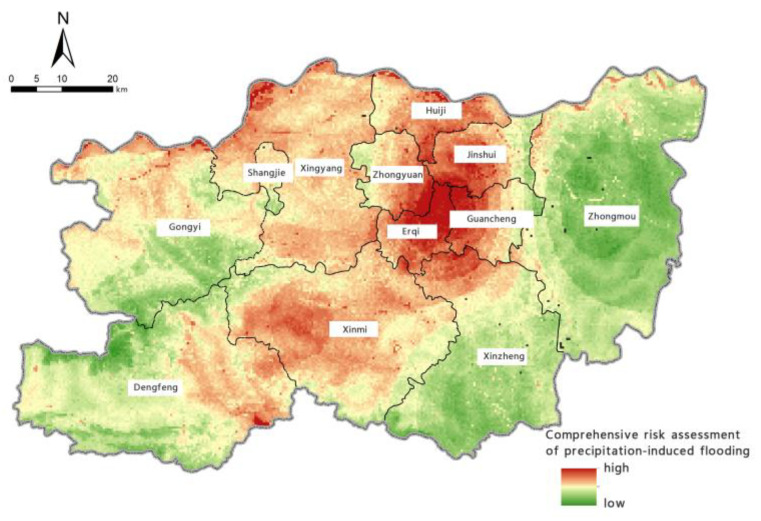
Map of the comprehensive risk assessment of precipitation-induced flooding.

The comprehensive assessment of the risk of precipitation-induced flooding in Zhengzhou was obtained by assigning weights to the four individual assessments of flood-aggravating environmental susceptibility, flood-causing risk, location-specific exposure, and flood-mitigation capability, as shown in Figure 10. The range of the comprehensive risk assessment is [0, 1]. The closer the value is to 1, the higher the risk of precipitation-induced flooding. It can be seen from Figure 10 that the risk of precipitation-induced flooding in Zhengzhou is high in central areas and low in eastern and western areas. Erqi District, Guancheng Hui District, Zhongyuan District, and Jinshui District in the central part of the city have the highest risk of precipitation-induced flooding, followed by Huiji District, Xingyang City, and Shangjie District in the north and Xinmi City in the south. Gongyi City and Dengfeng City in the west and Zhongmu County and Xinzheng City in the east have a relatively low risk of precipitation-induced flooding.

**Figure 11:**
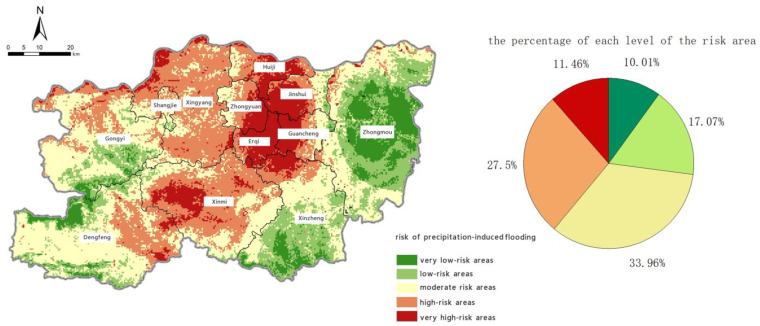
Zoning map of the risk of precipitation-induced flooding.

To help with regional precipitation-induced flooding prevention and control efforts, we divided Zhengzhou into very high-risk areas [0.700, 1.000], high-risk areas [0.420, 0.700], moderate-risk areas [0.245, 0.420], low-risk areas [0.165, 0.245], and very low-risk areas [0.100, 0.165]. The spatial distributions of these five levels are shown in Figure 11.

The total area of very high-risk areas in Zhengzhou is 866.81 km^2^, accounting for 11.46% of the city’s area. These are mainly in Erqi District, Guancheng Hui District, Jinshui District, Zhongyuan District, Huiji District, and the western part of Xinmi City as well as the northern part of Xingyang City near the Yellow River system. High-risk areas total 2081.18 km^2^ and account for 27.5% of the city’s total area. They are located around the very high-risk areas, mainly in Xinmi City in the central area, the northern parts of Xingyang City and Gongyi City, and the eastern part of Dengfeng City. Areas at moderate risk account for the largest area at 2569.64 km^2^, which is 33.96% of the city’s total area. They are the most widely distributed, including the 11 districts, counties, and county-level cities of Dengfeng City, Gongyi City, Shangjie District, Xingyang City, Xinmi City, Xinzheng City, Zhongmu County, Guancheng Hui District, Jinshui District, Zhongyuan District, and Huiji District. The spatial distribution of moderate-risk areas is strongly coupled with geographic factors, including elevation, slope angle, and land-use type. Low-risk and very low-risk areas cover 1292.25 km^2^ and 757.12 km^2^, respectively, and together they account for 27.09% of the total area of Zhengzhou. They are mainly in Gongyi City and Dengfeng City in the west of Zhengzhou and Zhongmu County and Xinzheng City in the east.

## 4. Discussion

### 4.1. Risk-Assessment Framework of Storm and Flooding Based on Four Indicators of Precipitation-Induced Flooding Risk

(1) The occurrences of storm and flood disasters are not independent events caused solely by sudden and heavy rainfall. Rather, the causes are often intertwined with external factors, including a region’s physical geography and socio-economic situation. Based on the “Natural Disaster Risk System”, this study conducted a census of all factors causing storm and flood disasters, and established a basic database of regional storm and flood disaster risks. The rainstorm and flood risk assessment model in Zhengzhou was effective in accurately assessing disaster risks from multiple perspectives.

(2) This study used machine learning algorithms to develop a storm and flood risk assessment system, overcoming traditional problems with indicator selection and weighting calculation processes being affected by the complex non-linear relationship between data and excessive subjective human influence. This work lays a foundation for further research applications of storm and flood risk assessments based on artificial intelligence and big data that will likely be of growing interest for the field of flood risk analysis.

(3) Drawing on the advantages of big data, remote sensing, global positioning systems (GPS), and geographical information systems (GIS), this study built on previous research using remote sensing of night-time light as an indicator, replacing the socio-economic factors of population density, urbanisation level, and per capita GDP. In doing so, it overcomes the disadvantage of excessive correlation between traditional socio-economic factors in such studies. At the same time, its grid-based data improved the accuracy of the evaluation results compared to traditional socio-economic factors that use the administrative region as a unit.

### 4.2. Strategies for Risk Management of Storm and Flood Disasters and Urban Planning in Zhengzhou

(1) Using the single variable maps and comprehensive risk assessment and zoning maps of the storm and flood risk in Zhengzhou, each district (city) can be managed according to its risk level.

The high-risk areas are mainly distributed in the Erqi, Guancheng Hui, Jinshui, Zhongyuan, and Huiji Districts, and the west of Xinmi City and north of Xingyang City, which are significantly affected by extreme precipitation. For these high-risk areas, cities should prioritise improving urban flood prevention emergency plans, strengthening real-time monitoring of precipitation and real-time evaluation, and improving the mechanism of information distribution regarding storms and floods. Simultaneously, as the populations and economies of these areas are relatively dense, improving public awareness of flood disaster prevention is particularly important [62]. Efforts should be channelled towards improving public opinion of the environment, promoting public participation in urban flood control emergency management [63] and exploring flood insurance, thereby boosting urban flood risk management capabilities.

The second-highest risk areas included the cities of Xinmi and Xingyang City, the northern part of Gongyi City, and the eastern part of Dengfeng City, which all experience location-specific exposure and flood-aggravating susceptibility.

These regions have developed fluvial systems and a high proportion of arable land. Therefore, the focuses for preventing storm and flood disasters should be flood control engineering and urban land-use planning, including: building flood walls and embankments along the river, strengthening existing flood control and drainage infrastructure, and preparing early flood warning and emergency plans. Furthermore, it is also important to strengthen land-use planning, management, and control, prioritise urban flood prevention, and coordinate and adapt urban construction to floods.

The eleven medium-risk areas include Zhongmu County; the cities of Dengfeng, Gongyi, Xingyang, Xinmi, and Xinzheng; and the Shangjie, Guancheng Hui, Jinshui, Zhongyuan, and Huiji Districts. These areas are moderately affected by flood-aggravating susceptibility, flood-causing risk, and location-specific exposures, but more importantly, they have a weak ability to prevent and reduce disasters. Therefore, emphasis should be placed on improving regional emergency management capabilities and enhancing public awareness of flood controls. On one hand, it is necessary to formulate emergency plans in large- and medium-sized cities, strengthen the allocation and coordination of manpower, and improve supervision and management to ensure timely early warnings reach every affected individual. On the other hand, the publication and education of meteorological information and emergency response capabilities [64] can be improved to enhance disaster prevention and self-rescue awareness.

The moderately low-risk and low-risk areas are mainly distributed in Zhongmu County, Xinzheng City, and the intersection of Gongyi Dengfeng Cities. Apart from public outreach and education, no other risk management is required.

(2) Under global warming, the frequency and intensity of extreme climate disasters have increased significantly for cities that are currently affected. Urban planning and construction are intrinsic to extreme climate risks. Multi-scale theoretical and practical research of disaster mitigation, adaptation, and planning is an important means of mitigating extreme climate disasters and improving urban resilience for the future. Using multiple forms of information technologies to conduct urban storm and flood disaster risk assessments, high-risk areas can be identified and urban flood control and drainage plans developed in advance. This is consistent with the three-step strategy of “assessment–warning–strategy”, and using flood control and risk reduction projects to manage flood disasters. Effectively restricting the construction and urban planning of lower-level sponge cities is beneficial to strengthening the blue and green lines, which represent water bodies and natural systems, management of ecological spaces, and vertical urban management. This method also allows for the optimisation of engineering decision-making, restricting or optimising projects and socio-economic development in risk areas, and improving early warning and forecasting.

## 5. Conclusions

Using the precipitation-induced flooding event that occurred in the city of Zhengzhou in the central Chinese province of Henan in July 2021 as a case study, we used the RF and XGBoost algorithms to examine the influencing factors and conduct a risk assessment of precipitation-induced flooding in Zhengzhou. Our research led to the following conclusions.

(1) Based on our evaluation of the geographical features of Zhengzhou and the quality of available data, we selected 16 indicators from the four aspects of geography, meteorology, population, and economy. We used RF to examine the contribution of each indicator to precipitation-induced flooding. We found that the four indicators with the highest contributions were (in descending order) total rainfall over three consecutive days, extreme daily rainfall, vegetation cover, and river systems. The indicators with the next highest contributions were land-use type, elevation, night-time light brightness, GDP, and slope angle.

(2) Based on the AUC and ACC of the RF model, we streamlined the indicators to create an optimised risk-assessment index. After removing the four indicators of economic growth, roads, slope aspect, and per capita GDP, which were the four bottom-ranked indicators in terms of contribution, the model’s prediction accuracy and performance were optimal.

(3) We used XGBoost to calculate the weights of the streamlined indicators for the final objective of constructing a risk-assessment model of precipitation-induced flooding in Zhengzhou to individually assess the four aspects of flood-aggravating environmental susceptibility, flood-causing risk, location-specific exposure, and flood-mitigation capability, which were integrated to obtain the final risk-assessment results of precipitation-induced flooding in Zhengzhou. The results showed that very high-risk and high-risk areas account for 11.46% and 27.50% of the total area of Zhengzhou, respectively. Their distribution is significantly affected by heavy rainfall, river systems, and land-use type. The areas with the highest risk of precipitation-induced flooding in Zhengzhou are Erqi District, Guancheng Hui District, Jinshui District, Zhongyuan District, Huiji District, and western Xinmi City.

(4) This study innovatively introduced a machine learning algorithm in the construction of a risk-assessment system for flooding; this overcomes the problem that the process of screening index factors and calculating weights is affected by complex non-linear relationships between data and excessive human subjective influence, improving the accuracy of the research results. This study can help government departments to identify high-risk areas and provide a scientific basis for storm and flood prevention and mitigation planning. However, there are still some limitations in this paper: (i) due to the limitation of data acquisition, only 18 basic factors were selected for secondary optimisation and screening, and it is proposed to expand the basic database in multiple ways in future studies and to continue to explore more comprehensive storm and flood hazard impact factors and their mechanism of action; (ii) due to the limitation of the original data type, some of the data were sourced from statistical tables and processed as panel data by the kriging difference method, resulting in the lack of precision of the data. In the future, we may consider and seek other raster data with higher accuracy for similar replacement.

## Figures and Tables

**Table 1:** Data sources of all indicators.

Primary Indicators	Secondary Indicators	Data Sources
Flood-aggravating environmental susceptibility	Elevation	Geospatial Data Cloud
	Slope angle	
	Slope aspect	
	River system	National Catalogue Service for Geographic Information-1:1 million national public basic geographic information data (2021)
	Roads	
Flood-causing risk	3-day rainfall	Data crawling from the National Meteorological Information Center
	Extreme rainfall	
Location-specific exposure	Night-time lights	Earth Observation Group of the National Centers for Environmental Information under the National Oceanic and Atmosphere Administration (US)
	Land use	Esri, using Sentinel-2 remote sensing images combined with an AI land classification model
	Arable land	
	Gross regional product	Zhengzhou Municipal Bureau of Statistics
	Gross domestic product per capita	
	Economic growth	
	Elderly population	
	Vegetation cover	Calculated based on remote sensing data from the Landsat-8 satellite in 2021
Flood-mitigation capability	Medical rescue services	Data on general hospitals and 3A hospitals from Baidu Maps

**Table 2:** Experimental process record sheet.

Experiment	Deleted Indicator(s)	ACC	AUC
1	None	0.880111	0.948667
2	Economic growth	0.889333	0.955333
3	Economic growth, roads	0.887667	0.961889
4	Economic growth, roads, slope aspect	0.906111	0.966333
5	Economic growth, roads, slope aspect, GDP per capita	0.913333	0.967111
6	Economic growth, roads, slope aspect, GDP per capita, medical response	0.900000	0.967333
7	Economic growth, roads, slope aspect, GDP per capita, medical response, elderly population	0.891111	0.962778
8	Economic growth, roads, slope aspect, GDP per capita, medical response, elderly population, proportion of arable land	0.887333	0.957667

GDP: gross domestic product; ACC: accuracy; AUC: area under the curve.

**Table 3:** Index weights for precipitation-induced flooding risk assessment.

Target	Primary Indicators	Secondary Indicators	Weight	Indicator Type
Precipitation-induced flooding risk	Flood-aggravating susceptibility	(a) Elevation	0.085	Negative
		(b) Slope angle	0.054	Negative
		(c) River system	0.096	Positive
	Flood-causing risk	(d) 3-day rainfall	0.146	Positive
		(e) Extreme daily rainfall	0.188	Positive
	Location-specific exposure	(f) Night-time lights	0.045	Positive
		(g) Land use	0.103	Positive
		(h) Arable land proportion	0.064	Positive
		(i) GRP	0.032	Negative
		(j) Elderly population	0.020	Positive
		(k) Vegetation cover	0.138	Negative
	Flood-mitigating capability	(l) Medical response	0.028	Negative

## Data Availability

No new data were created or analysed in this study. Data sharing is not applicable to this article.
